# Risk-based strategies for surveillance of tuberculosis infection in cattle for low-risk areas in England and Scotland

**DOI:** 10.1017/S0950268817001935

**Published:** 2017-12-06

**Authors:** L. C. M. SALVADOR, M. DEASON, J. ENRIGHT, P. R. BESSELL, R. R. KAO

**Affiliations:** 1 Boyd Orr Centre for Population and Ecosystem Health, Institute of Biodiversity, Animal Health and Comparative Medicine, College of Medical, Veterinary and Life Sciences, University of Glasgow, Glasgow, UK; 2 The Roslin Institute, The University of Edinburgh, Easter Bush, Midlothian, UK; 3 Computing Science and Mathematics, Faculty of Natural Sciences, University of Stirling, Stirling, UK

**Keywords:** Bovine tuberculosis, risk factors, risk-based surveillance, routine-herd-testing, slaughterhouse

## Abstract

Disease surveillance can be made more effective by either improving disease detection, providing cost savings, or doing both. Currently, cattle herds in low-risk areas (LRAs) for bovine tuberculosis (bTB) in England are tested once every 4 years. In Scotland, the default herd testing frequency is also 4 years, but a risk-based system exempts some herds from testing altogether. To extend this approach to other areas, a bespoke understanding of at-risk herds and how risk-based surveillance can affect bTB detection is required. Here, we use a generalized linear mixed model to inform a Bayesian probabilistic model of freedom from infection and explore risk-based surveillance strategies in LRAs and Scotland. Our analyses show that in both areas the primary herd-level risk factors for bTB infection are the size of the herd and purchasing cattle from high-risk areas of Great Britain and/or Ireland. A risk-based approach can improve the current surveillance system by both increasing detection (9% and 7% fewer latent infections), and reducing testing burden (6% and 26% fewer animal tests) in LRAs and Scotland, respectively. Testing at-risk herds more frequently can also improve the level of detection by identifying more infected cases and reducing the hidden burden of the disease, and reduce surveillance effort by exempting low-risk herds from testing.

## INTRODUCTION

Bovine tuberculosis (bTB) in cattle is the most economically important disease of livestock in Great Britain (GB), with substantial impact on animal health and welfare, farmers’ livelihoods and their well-being [1–6]. A zoonosis, bTB is caused by *Mycobacterium bovis* and it affects an increasing number of cattle herds in GB, with an especially high incidence in the South West of England and in Wales (high-risk areas or HRAs) [4, 7]. While factors such as herd size, being a dairy farm, and some farming practices have been identified as herd-level risks [3, 8–13], the presence of a wildlife reservoir, the Eurasian badger (*Meles meles*), makes the control of the disease particularly difficult and controversial [2, 11, 14–16]. In contrast, wildlife reservoirs do not appear to make a meaningful contribution to the spread of bTB in cattle into low-risk areas (LRAs) such as the north and east of England, or into Scotland [4, 17]. While large outbreaks in cattle do occur here [18] and the risk of establishment in wildlife must be considered, in these areas control efforts can focus on infection in cattle, and particularly on imports from HRAs and from Ireland (both the Republic of Ireland and Northern Ireland), where bTB prevalence is also high [3, 12, 18–20]. The prevalence of bTB in LRAs and in Scotland has been stable since 2006 and it remains at low levels (~0·1 in LRAs and <0·01 in Scotland) [17]. While other supplementary tests are used [21, 22], bTB cases are mostly identified using the Single Intradermal Comparative Cervical test (SICCT or colloquially, the ‘skin test’) or by carcass inspection at the slaughterhouse. A positive reaction is defined as a difference of >4 mm in skin swelling when comparing responses with antigenic derivatives from *Mycobacterium avium* and *M. bovis*, 72 h post injection. An animal with a positive test (reactor) is slaughtered and movement restrictions are imposed on the farm. A reactor is then confirmed by identification of at least one tuberculous lesion *post-mortem* or if *M. bovis* is successfully cultured from tissue samples.

Traditionally, the bTB surveillance in GB regime allowed for different frequencies of routine herd testing (1 year RHT: HRAs, 2–3 year RHT: intermediate risk areas, 4 year RHT: LRAs), which was based upon the perceived risk of bTB in a given parish or the neighbouring parishes. In recent years more geographically streamlined designations have been introduced. All herds in Wales were officially placed on annual RHT in 2010. In England, a new bTB surveillance regime has been in place since 1 January 2013, whereby most herds in counties of the so-called low-risk area (LRA) are tested once every 4 years, whereas herds in the HRA of the West of England and a ‘transitional’ zone of intermediate bTB incidence known as the Edge Area (currently encompassing Hampshire, Berkshire, Buckinghamshire, Northamptonshire, Leicestershire, Nottinghamshire and parts of East Sussex, Oxfordshire, Warwickshire, Derbyshire and Cheshire) are tested annually (see online Supplementary Fig. S1). In order to speed up the detection of infected herds in increasing bTB incidence parts of the Edge Area, a 6-monthly testing regime has been established since January 2015. Furthermore, at the end of 2016, Defra consulted on proposals to replace annual testing with 6 monthly herd testing in other high bTB incidence parts of the Edge Area of England. This change in the surveillance testing regime may come into effect during 2017. Additional targeted testing is also performed in herds contiguous to herds with Officially Tuberculosis Free Status Withdrawn (OTFW) due to fully confirmed infection (contiguous and radial tests), in herds that have recently regained Officially TB Free Status after suffering an episode of bTB (6- and 12-monthly check tests), herds with back traced reactors or cases detected through slaughter surveillance (immediate check tests), herds in annual testing areas that send animals to other farms (pre-movement tests) and herds in LRAs that receive animals from annual testing areas (post-movement tests) or from Ireland (post-Irish imports).

In Scotland, all eligible herds were tested every 4 years under the RHT policy until 31 December of 2011; since then, a risk-based surveillance system has been adopted exempting from RHT herds identified as low risk and with high chance of detection via abattoir inspection, offering a cost reduction by requiring fewer herd and animal tests, and fewer false positives [23]. We hypothesise that, similar to Scotland, the current surveillance system in LRAs could be enhanced by a more targeted, risk-based approach that either reduces testing of cattle (thereby saving cost and effort), or enhances identification of infection (thereby reducing onward risk), or does both. Based upon this, the objectives of this work are to (1) identify risk factors associated with breakdowns in these areas; (2) use a mixed logistic regression statistical model to compute the herd probability of freedom of infection; and (3) use a risk-based surveillance model to evaluate alternative surveillance strategies.

## MATERIALS AND METHODS

### Underlying risk factors for bTB breakdowns

#### Source data

Analyses were performed using data between 2007 and 2013 from active holdings (determined by the unique County-Parish-Holding or CPH number, with all cattle associated with that holding assumed here to belong to one ‘herd’) in LRAs and in Scotland. Data on the historical SICCT test interval were used to identify herds in LRAs in England (on 4 yearly testing throughout the period considered), since the number of these herds has decreased over time (online Supplementary Fig. S1).

Data on holdings, on historic bTB test results, on incidence and on animal life histories are taken, respectively, from the Defra animal health information system (SAM) provided by the Animal & Plant Health Agency (APHA) and from the British Cattle Movement System (BCMS) Cattle Tracing System Database (CTS). Data extraction were done as described in online Supplementary Section S2.1, which resulted in analyses on 13 327 herds in LRA and 10 145 herds in Scotland.

#### Statistical analysis

Multivariate logistic mixed models were formulated to assess bTB candidate risk factors in herds in Scotland and in England (with 4-year testing) during 2008 and 2013. Both year and county were included as random effects and annual mean herd size, herd type (according to online Supplementary Table S1), Irish imports and high-risk movements were considered as fixed effects (see online Supplementary Section S2.2 for more details). Models were evaluated using the AIC model score according to online Supplementary Section S2.3. Only the predictors that were significant at *P* < 0·05 were included in the final model. Interactions and associations between variables were also explored. The significant fixed effects are presented as odds ratios (Odds) with 95% confidence intervals. The fitted values extracted from the most significant model correspond to the probability of the herd becoming infected in each year of the study (*p*_*intro*_).

The data were prepared using a combination of UNIX bash scripting and AWK commands for data extraction, and R [24] for data processing and management. The multivariate logistic mixed models and the model selection were developed in the R lme4 [25, 26] and LMERConvenienceFunctions [27] packages.

### BTB surveillance framework

#### Source data

Using the same study period as above, per herd data were extracted from the SAM and CTS databases and derived from the risk factor analysis (Section ‘Underlying risk factors for bTB breakdowns’). Batches of cattle in the previous year from holdings to slaughterhouse were extracted from CTS. Holdings that did not send any animals to the slaughterhouse were not considered, resulting in a total of 12 942 herds from 13 327 low-risk English herds and 9776 herds from 10 145 Scottish herds used in the analysis in the section ‘Underlying risk factors for bTB breakdowns’. The following variables were calculated for each herd for every year between 2008 and 2013: herd size, herd type, herd-level prevalence of infection, number of batches from HRAs received in the previous year, number of Irish imports received in the previous year and number of batches sent to slaughterhouse in the previous year.

#### Surveillance scenarios

A number of baseline and risk-based surveillance options were explored, considering both the likelihood of becoming infected and detected by SICCT and at the slaughterhouse. The baseline scenarios include slaughterhouse surveillance without live testing of herds, which represents a minimal model (the lowest amount of surveillance that a herd could be under) or, in addition, either 1-year (the maximal model that corresponds to the most prevalent surveillance approach in HRAs under the current testing regime), 2-year or 4-year RHT (the models that correspond to RHT surveillance every 2 or 4 years, respectively).

The risk-based surveillance scenarios were modelled considering the risk factors identified in the section ‘Underlying risk factors for bTB breakdowns’ together with the proportion of the herd's total stock that is sent annually to slaughter as elements of infection risk and detection. All modelled risk-based scenarios also include routine slaughter surveillance (baseline). Table 2 provides a summary of the surveillance components of each scenario. ‘Scenarios 1–4’ test more often herds that import ‘high-risk’ animals and send a low proportion of the total herd to slaughter every year; ‘scenarios 5–6’ test more often herds that import ‘high-risk’ animals, send a low proportion of animals to slaughter, and that have a large number of animals; ‘scenarios 7–8’ test more often herds that only have a large number of animals, and, the ‘Scottish scenario’ represents the surveillance that has been implemented in Scotland since 1 January 2012, in which herds that have a large number of animals, import ‘high-risk’ animals and send a low number of animals to slaughter are not exempt from testing. A detailed description of the elements of risk of infection, of the model assumptions and of the risk-based scenarios point-score system can be found in online Supplementary Section S3.

#### Model description

To evaluate the likelihood of herd-level freedom from infection (herd probability of undetected infection) with bTB during a specified time period (*t*) the model requires the following parameters:

- The probability of the herd becoming infected at time *t* (*p*(*intro*)_*t*_) (from the section ‘Underlying risk factors for bTB breakdowns’);
- Herd size at time *t*;
- The efficacy of the surveillance system implemented on the farm (RHT and slaughterhouse testing)
- The herd-level prevalence of infection *p*_*star*_.

The model only considers explicitly RHT and slaughterhouse surveillance. The efficacy of the surveillance system is evaluated by calculating the herd-level test system sensitivity *se*_*system*_, which includes the RHT and part slaughterhouse testing:

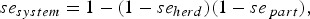
in which *se*_*herd*_ is the sensitivity of the SICCT implemented as a herd test, and *se*_*part*_ is the part herd sensitivity for slaughterhouse surveillance. The herd sensitivity for a whole herd test is calculated as

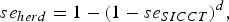


Where *se*_*SICCT*_ is the animal-level sensitivity of the screening test and *d* is the number of infected animals in the herd defined as:

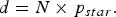
The value *d* is the product of a number drawn from the distribution of *p*_*star*_ and the annual average number of animals in the herd *N*. The sensitivity for a part herd test for the proportion of the herd that is sent to slaughter is:

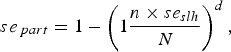
where *n* is the number of animals sent to slaughter per annum.

The false positive (unconfirmed breakdown) detection rate of the whole herd test *sp*_*herd*_ is defined as:

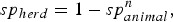
where 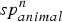 with *n* = *N* is the specificity for whole herd tests.

In this analysis, the values used for the test sensitivities and specificities follow the meta-analysis of bTB diagnostic test performance in Great Britain performed by Downs *et al*. [28].

The probability of freedom (the posterior) at time *t* is given by:

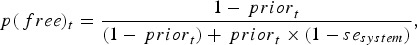
where *prior*_*t*_ is the prior probability that the herd is infected at time *t*. The prior for *t* + 1 is:

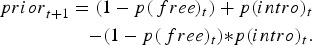


#### Model evaluation

The model was implemented in the R statistical environment and run for 100 simulations using data between 2008 and 2013, with 2008 used as a ’burn-in’ period. The start of the routine herd testing cycle (e.g. every 1-, 2-, 4-years) was generated randomly for each simulation, to minimise cyclical effects. The risk-based scenarios were evaluated from 2009 to 2013 by comparing the following variables to the equivalent fitted values from the current surveillance scenarios in each area (LRA: 4 year-RHT, Scotland: Scottish scenario; the national totals for each term are given by summing the values for all herds). If the absolute difference between two values is <5%, then these values are considered similar. Derivation of each term for each holding at a time t is described below:

- The annual expected number of latent infections. Probability of being latently infected for each farm at time t: (1 − *p*(*free*)_*t*_).
- The total fitted number of detected infections. Probability of a detected infection at time: (*prior*_*t*_ − *p*(*free*)_*t*_).
- The annual number of herds tested.
- The annual number of cattle tested.
- The annual expected number of false positives. The parameter *sp*_*herd*_ gives the probability of a given herd being a false positive. Therefore, summing up *sp*_*herd*_ for all herds for a given year gives the expected number of false positives.

A scenario is considered to have improved detection if it has similar or higher number of detections and lower number of latent infections.

#### Comparison of bTB risk between LRAs and Scotland

To compare the risk of bTB infection between LRAs and Scotland, we have applied the predictor of the best-fitted statistical model in LRAs to Scottish herds, and the predictor of the best-fitted model in Scotland to the English herds (see the section ‘Underlying risk factors for bTB breakdowns’). Should LRA herds with the same predicted probabilities under the Scottish model tend to have higher breakdown rates (or vice versa) this would imply an inherently different epidemiological risk between these areas, for example different demographics of the respective cattle populations, presence of a wildlife reservoir or any other risk factor not captured by these models.

## RESULTS

### Underlying risk factors for bTB breakdowns in LRAs and Scotland

**Fig. 1.**
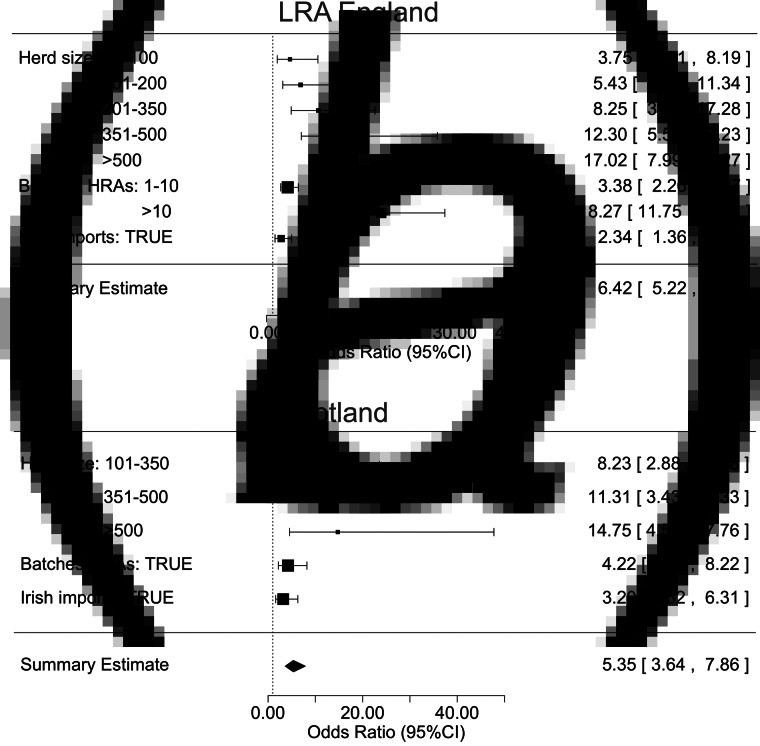
The results of a mixed logistic regression model that computes the herd risk of infection and determines the risk factors associated with bovine Tuberculosis breakdowns in low-risk areas in England (a) and in Scotland (b) between 2008 and 2013. Odds ratios and 95% confidence intervals express the contribution of each one of the significant risk factors.

The models created for the risk-factor analysis have a substantial predictive value, as indicated by the area under the curve (LRA England, AUC = 0·8685; Scotland, AUC = 0·8533; online Supplementary Fig. S2). The significance of the variables used to determine the risk factors in LRAs and in Scotland was estimated for the study period 2008–2013. Of the variables considered in the analysis, herd size, receiving batches from HRAs, and Irish imports were associated with bTB incidents in both LRAs and Scotland (Fig. 1, online Supplementary Table S2). The larger the herd, the higher is the association with bTB infection (Fig. 1 and online Supplementary Fig. S3 and Table S2). This association also applies across different herd types (online Supplementary Fig. S4), with the exception of dairy herds where there is a large overlap of the two distributions, and of store herds (young beef or mixed-breed animals reared and sold for finishing on fattening farms) in Scotland, which did not suffer any breakdowns (online Supplementary Fig. S4-B).

Receiving batches from HRAs and/or importing animals from Ireland in the previous year are also associated with bTB incidents in both areas (Fig. 1, online Supplementary Table S2). This outcome is consistent with an independent analysis of movements as risks (online Supplementary Section S1, Fig. S5).

### BTB surveillance in LRAs and in Scotland

#### Probability of undetected infection

**Fig. 2.**
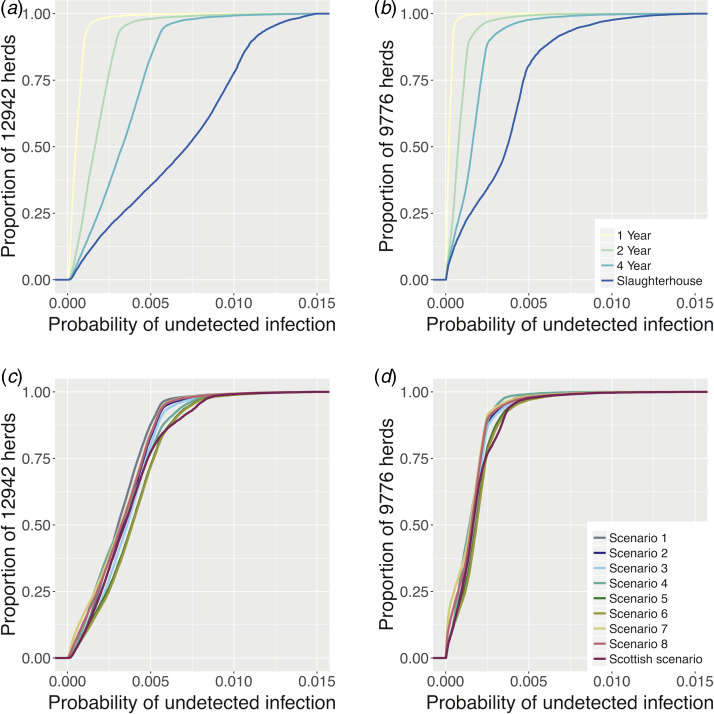
The cumulative distribution plot of the probability of each herd harbouring undetected infection at the end of each model time step for all eligible herds in LRA England (a) and in Scotland (b) between 2009 and 2013 from the four baseline surveillance scenarios. Subplots C and D show the cumulative distribution plot of the probability of each herd harbouring undetected infection at the end of each model time step for all eligible herds in LRA England (c) and in Scotland (d) during 2009–2013 from nine risk-based surveillance scenarios. The axes were truncated for clarity.

In general, the probability of undetected infection per herd in LRAs is higher than in Scotland for all the presented scenarios (Fig. 2). While the probabilities under different baseline scenarios are distinctly different, these differences are substantially reduced for the risk-based schemes, especially in Scotland where some of the curves are overlapping. As expected, the high probability with slaughterhouse surveillance alone (grey line, Fig. 2a,c) decreases with increased RHT.

Finishing herds have a lower probability of undetected infection (higher probability of freedom from infection) in both LRAs and in Scotland (online Supplementary Fig. S6). In LRAs, they are followed by dairy, by both beef and suckler, and by store, while for the period of the study, Scotland did not register any breakdowns in store herds (online Supplementary Fig. S4-B), making beef and suckler (herd of cattle composed of dams and their young calves up to the point of weaning) the riskiest herds, followed by dairy. There is a linear relationship between the average number of animals sent to slaughter per year and the average herd size with distinct clustering of finishing and dairy herds both in LRAs and in Scotland (online Supplementary Fig. S7). In LRAs, finishing herds are typically smaller than dairy herds (with the exception of some outliers), however, they have more per capita movements to slaughter than dairy ones, followed by store, beef and suckler (online Supplementary Fig. S7-a). In Scotland, the trend is the same as in LRAs, with the exception that suckler herds have more per capita movements to slaughter than beef and dairy (online Supplementary Fig. S7-b).

When herd-level risk of infection is incorporated into surveillance scenarios, the cumulative herd level probabilities of harbouring undetected infection (calculated by the cumulative distribution function (CDF)) are very similar across the different risk-based scenarios (Fig. 2), reflecting the importance of slaughterhouse surveillance for many herds (the one factor that is constant across scenarios). In LRAs, for higher values of proportion of herds tested, scenario 1 is the one that shows the lowest probability of undetected infection per herd, followed by scenarios 7 and 8. In Scotland, there are no major differences between the different scenarios, with the exception of scenarios 5, 6 and Scottish scenarios, which present slightly higher probabilities of harbouring undetected infection per herd.

#### Surveillance scenarios ranking

**Fig. 3.**
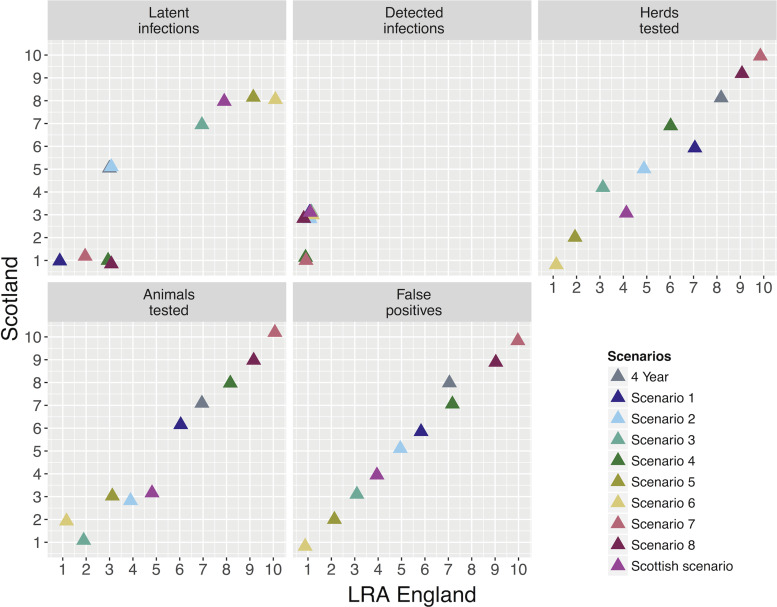
Risk-based surveillance scenarios comparison between LRA England and Scotland. Each panel represents the surveillance variables used in the probability of freedom model and the *x*- and *y*-axes represent the rankings of each scenario. Each colour represents the different scenarios and the triangle overlap represents that more than one scenario had the same ranking related to the variable in study. The 4-year scenario was also included in the analysis for comparison with the risk-based ones.

The different surveillance scenarios were ranked according to their levels of detection (numbers of latent and detected infections), testing burden (numbers of animals and herds tested annually) and the number of SICCT false positive herd results generated. The values of these variables were estimated for each scenario for both areas, were compared with the current surveillance regimes in each area (LRAs, ‘4 year RHT’; Scotland, ‘Scottish scenario’) and are presented in Figure 3 and in Table 1. Baseline scenarios perform as expected, with the notable feature that increased frequency of RHTs (Table 1) greatly reduce the number of latent infections with RHT frequency (4 and 15 compared with 32 in LRAs; 1 and 5 compared with 30 in Scotland), but with a minimal increase in the number of detected infections, while generating substantially more false positives (2 and 4 times more than the current systems).

**Table 1.** Summary of the bovine TB surveillance analysis results for low-risk areas in England and for Scotland between 2009 and 2013

Surveillance scenario	Interval (years)	Herds tested		Animals tested		Detected infections		Latent infections		False positives	
		LRA	Scotland	LRA	Scotland	LRA	Scotland	LRA	Scotland	LRA	Scotland
Baseline scenarios											
Slaughterhouse	n.a.	0	0	0	0	120	33	80	30	0	0
4 Year	4	3235	2444	391 613	412 293	144	45	32	12	72	58
2 Year	2	6471	4888	783 226	824 587	142	46	15	5	143	115
1 Year	1	12 942	9776	1 566 452	1 649 175	136	45	4	1	286	231
Risk-based surveillance											
Scenario 1	0/2/4	3065	2090	369 673	349 453	147	45	29	11	68	49
Scenario 2	0/4	2531	1930	290 579	304 968	143	45	33	12	56	45
Scenario 3	0/4	2074	1629	220 621	227 379	142	44	35	13	45	37
Scenario 4	0/1/2/4	2827	2262	448 135	454 829	143	46	33	11	68	57
Scenario 5	0/1/2/4	1803	1483	264 090	308 083	139	43	39	14	41	36
Scenario 6	0/1/2/4	1682	1328	213 163	244 088	138	42	40	14	37	31
Scenario 7	1/4	3878	3428	735 237	1 352 799	145	46	30	3	270	207
Scenario 8	1/4	3486	2871	570 664	736 476	144	45	31	11	80	72
Scottish scenario	0/4	2166	1602	292 074	306 499	139	43	37	14	51	41

The ‘4 year’ baseline scenario is the one currently implemented in LRAs and the ‘current’ risk-based scenario is the one that has been implemented in Scotland since January 2012.

Results of the risk-based surveillance model (all figures per annum) are presented in Figure 3 and Tables 1 and 2. These can be divided into three different categories, all of which reduce the burden of testing:

1. Scenarios that improve detection
  In LRAs, scenario 1 decreases latent infections by 9% (29 compared with 32) for a similar level of detected infections, it tests 5% fewer herds annually (3065 compared with 3235), 6% fewer animals (369 673 compared with 391 613), and generates 6% fewer false positives (68 compared with 72), when compared with the current 4-Year RHT regime. In Scotland, scenario 2 reduces latent infections by 7% (12 compared with 14) for a similar number of detected cases (within 5%), tests 26% fewer animals (304 968 compared with 306 499 for a slight higher number of herds tested) and generates 10% fewer false positives (45 compared with 41) than the current surveillance regime (Scottish scenario).
2. Scenarios that reproduce similar levels of detection.
  For the same level of detection, *scenario 2* offers a saving of 22% on the number of herds tested annually (2531 compared with 3235), 26% on the number of animals tested (290 579 compared with 391 613), and 24% on the number of false positives (56 compared with 72) in LRAs when compared with the current 4-year RHT regime. In Scotland *scenario 6* reduces the herd testing by 17% (1328 compared with 1602), the animal testing by 20% (244 088 compared with 306 499), and the number of false positives by 24% (31 compared with 41) when compared with the Scottish scenario.
3. Scenarios that substantially reduce the burden of testing but lower the levels of detection.
  The scenario that offers savings on the burden of testing but at the expense of reduced detection of infected herds in LRAs is scenario 3. This scenario would reduce the testing burden by testing 44% fewer animals (220 621 compared with 391 613), 36% fewer herds (2074 compared with 3235) and generate 38% fewer false positives (45 compared with 72) while increasing the number of latent infections by 9% (35 compared with 32) for the same level of detection. This scenario would also miss 3 out of 13 breakdowns identified by RHT (Table 2).

**Table 2.** Composition of the risk based surveillance scenarios for low-risk areas in England and Scotland during 2009–2013

Criteria	Points	Testing interval	No. herds (%)		bTB cases		RHT	
			LRA	Scotland	LRA	Scotland	LRA	Scotland
Scenario 1								
Slaughtering <25% of stock	+1	0 points = no testing	2632 (20·3)	2015 (20·6)	93	18	2	2
Receiving ‘high-risk’ animals in >3 years and slaughtering <50% of stock	+1	1 point = 4 year testing	8359 (64·6)	7159 (73·2)	34	22	10	9
		2 points = 2 year testing	1951 (15·1)	602 (6·2)	19	5	6	2
Scenario 2								
Slaughtering <25% of stock and/or receiving ‘high-risk ’ animals in >3 years and slaughtering <40% of stock	1	0 points = no testing	2815 (21·8)	2053 (21)	94	19	2	2
		1 point = 4 year testing	10 127 (78·2)	7723 (79)	52	26	16	11
Scenario 3								
Slaughtering <12·5% of stock and/or receiving ’high-risk’ animals in >3 years and slaughtering <25% of stock	1	0 points = no testing	4643 (35·9)	3259 (33·3)	106	26	4	3
		1 point = 4 year testing	8299 (64·1)	6517 (66·7)	40	19	14	10
Scenario 4								
Slaughtering >25% of stock	−1	−1 or 0 points = no testing	4524 (35)	3152 (35)	27	12	4	3
Slaughtering <5% of stock	+1	1 point = 4 year testing	6166 (47·6)	4585 (46·9)	101	22	8	4
Receiving ’high risk’ animals in >3 years	+1	2 points = 2 year testing	1932 (14·9)	1845 (18·9)	14	10	6	4
Having >100 animals	+1	3 points = 1 year testing	320 (2·5)	194 (2)	4	1	0	2
Scenario 5								
Slaughtering >25% of stock	−1	−1 or 0 points = no testing	6828 (52·8)	4504 (46·1)	72	25	8	4
Slaughtering <5% of stock	+1	1 point = 4 year testing	5076 (39·2)	4679 (47·9)	67	15	9	6
Receiving ’high risk’ animals in >3 years	+1	2 points = 2 year testing	1007 (7·8)	559 (5·7)	7	5	1	3
Having >350 animals	+1	3 points = 1 year testing	31 (0·2)	34 (0·3)	0	0	0	0
Scenario 6								
Slaughtering >25% of stock	−1	−1 or 0 points = no testing	7160 (55·3)	4878 (49·9)	89	31	11	6
Slaughtering <5% of stock	+1	1 point = 4 year testing	4859 (37·5)	4499 (46)	50	10	6	5
Receiving ’high risk’ animals in >3 years	+1	2 points = 2 year testing	910 (7)	390 (4)	7	4	1	2
Having >500 animals	+1	3 points = 1 year testing	13 (0·1)	9 (0·1)	0	0	0	0
Scenario 7								
Having ⩽350 animals	1	1 point = 4 year testing	857 (6·6)	1312 (13·4)	58	14	3	4
Having >350	3	3 points = 1 year testing	12 085 (93·4)	8464 (86·6)	88	31	15	9
Scenario 8								
Having ⩽500 animals	1	1 point = 4 year testing	12 607 (97·4)	9206 (94·2)	110	37	17	11
Having >500	3	3 points = 1 year testing	335 (2·6)	570 (5·8)	36	8	1	2
Scottish scenario								
Having >20 animals and receiving ‘high-risk’ animals more than once in the last 4 years and/or slaughtering >25% of stock and receiving ‘high-risk’ animals more than once in the last 4 years and/or slaughtering >40% of stock	1	1 point = 4 year testing	8665 (67)	6410 (65·6)	54	27	1	2
		0 points = no testing	4277 (33)	3366 (34·4)	92	18	17	11

The testing interval column represents the time frame of the bTB testing, which depends on the level of risk based on a score point system (0 = no testing, 1 = 4 year testing, 2 = 2 year testing, 3 = annual testing). The No. herds column corresponds to the number of herds (and percentage of the total number of herds) that fell into each testing interval category. bTB is the number of confirmed breakdowns for the eligible herds between 2009 and 2013. RHT column represents the breakdowns that were detected by routine-herd testing.

The remaining scenarios would increase the burden of testing with at best minimal detection benefits, and therefore would not contribute to an increased performance of the surveillance system and are not considered further here.

#### Comparison of bTB risk between LRAs and Scotland

**Fig. 4.**
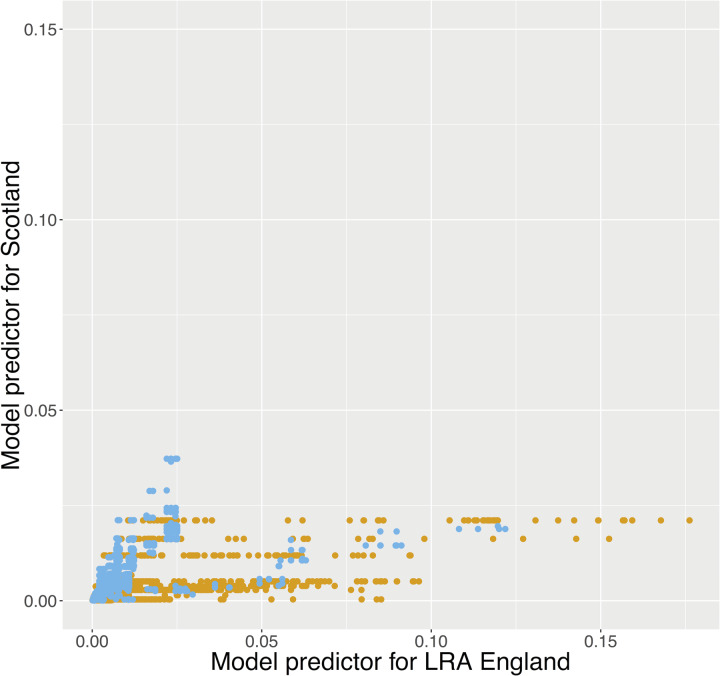
Herd probability of infection given by the English (LRA) and Scottish predictors. The different colours represent the location of each herd (orange: LRA England, light blue: Scotland). If herds in Scotland were exposed to the same level of risk as herds in LRA England, their probability of getting infected would be higher. From the other hand, if herds in LRA England were exposed to the same level of risk as herds in Scotland, their probability of getting infected would be lower.

The herd probability of infection given by the English and Scottish predictors is presented in Figure 4. The estimated herd probability of bTB infection is lower in Scotland than in LRAs (*μ*(Scot) = 0·00080, *σ*^2^(Scot) = 2·5e-06; *μ*(LRA) = 0·0020, *σ*^2^(LRA) = 4·1e-05; online Supplementary Fig. S8) and if Scottish herds were exposed to the same risk as herds in LRAs the average risk per herd would increase to 0·0013 (*σ*^2^ = 7·2 × 10^−6^, online Supplementary Fig. S9). However, if LRA English herds were exposed to the same risk as herds in Scotland, the predicted risk of infection would be similar to Scotland (*μ* = 0·00080, *σ*^2^ = 2·3 × 10^−6^) suggesting that there is an inherent geographical difference between the two areas, with the risk of bTB infection higher in LRAs.

## DISCUSSION

The development of our proposed risk-based surveillance strategy has two major aims. The first is to identify inefficiencies: in the case of bTB, this amounts to identifying where slaughterhouse surveillance works well and therefore RHT can most safely be discarded. The second is to identify those herds most likely to present an infection risk: both those most likely to become infected and (more difficult to define) those more likely to present risks to others. For bTB in the absence of a wildlife reservoir, larger herds and those that purchase cattle from HRAs are more likely to become infected, while herds that sell on many cattle to other premises, present the greatest infection risk. As our statistical models do not capture this onward transmission related risks, and as some of these risks are captured indirectly by herd characteristics, intelligent decision-making is sometimes required to balance off these issues. For example, finishing herds bring in many cattle and therefore are a high risk of becoming infected, however they also send many cattle to slaughter and do not sell on many cattle to other premises, and therefore present a low risk to others.

Despite some broad similarities (the same risk factors and some similarities in the cattle industry), risks compared across LRAs and Scotland have some subtle differences, notably the mean risk of infection of bTB per herd (LRAs have higher probability of risk of infection per herd than Scotland), and differences in per capita movements to slaughter (in LRAs, beef herds have more per capita movements to slaughter than suckler and dairy herds, while in Scotland suckler herds have more per capita movements to slaughter than beef and dairy). Store herds have the lowest probability of freedom from infection with slaughterhouse surveillance only, while in Scotland they are the least risky after finishing herds.

England and Scotland have proceeded along different surveillance paths until now, mainly as a result of differences in the underlying legislative and policy environments. As the EU granted official bTB free (OTF) status to Scotland in September 2009, this offered the opportunity to do away with routine testing of low-risk herds (while maintaining very similar detection rates), and so a risk-based surveillance strategy based on our previous work [23] was adopted. The evidence presented here suggests some refinements that could offer a higher reduction on the testing burden while maintaining the current level of bTB detection in Scotland (scenarios 2 and 6). By contrast, in the LRA of England the process to secure OTF status has already begun, but has not yet been achieved. Therefore, any reduction of testing at this stage would present a greater risk and could jeopardise this process. However, it is something that policymakers should consider if the LRA is recognised as OTF in due course. On the other hand, the present study shows that there is little to be gained from increased routine herd testing frequencies in the LRA. Whether or not changes in surveillance in LRAs are currently merited, nonetheless it is important to understand the extent to which the areas considered low risk in England may differ from Scotland, as this may be an indicator of the feasibility of achievement of OTF status in LRAs.

A good prediction of the risk of infection in both LRAs and in Scotland is determined by the size of the herd, by movements from HRAs, and by whether herds receive animals from Ireland. Larger herds have a greater likelihood of infection in both areas, confirming previous results in an earlier time period [4, 29]. The continued significance of the high-risk movements despite pre-movement testing suggests that greater test sensitivity could accrue substantial benefits (possibly even if there is a cost of reduced test specificity). The risk of bTB breakdowns can also be due to local effects such as the history of bTB cases in the herd [4, 19] and poor farm biosecurity [30]. These factors have not been considered in the analysis, but it is likely that they also play a role and be responsible for the persistence and spread of the disease. That herds in LRAs have substantially higher risk of infection than in Scotland even under the same risk model is notable, suggesting that there are important epidemiological factors not captured in our models; this merits further study beyond the scope of this paper.

In this work, a model of freedom from infection was used to evaluate different strategies for surveillance for bTB in LRAs and Scotland. By exploiting statistical analyses defining herd level probability of freedom from disease layered by significant risk factors, testing frequencies can be refined to test highly probable infections more frequently, and conversely potentially reducing testing of low-risk herds. The strategies modelled here focused on significant indicators of risk, penalizing herds with large sizes that receive ‘risky’ imports (movements from HRAs and Ireland). Herds that send a small proportion of their stock to slaughterhouse are also penalised, as this makes slaughterhouse detection less likely.

While the actual policy implemented will depend on other considerations such as the practicality of implementation and the differential impact on the various sectors of the industry, our analyses show that the current schemes in LRAs and in Scotland are effective and a more frequent testing regime (1 or 2 years interval) will provide higher freedom from disease (originate fewer latent infections), but will not improve the current number of detected breakdowns and will substantially increase the burden associated with testing. However, surveillance in LRAs can be improved if herds that are at higher risk are targeted. It is not expected that these factors affect the performance of the model, however. As the framework used is scalable and adaptable, it can be extended with other surveillance scenarios, as well as other policy measures currently implemented in both LRAs and Scotland, such as pre-movement and post-movement testing.

## CONCLUSION

This paper has demonstrated that the determinants of bTB infections in LRAs of England and in Scotland are very similar to each other, but that the magnitude of risk in LRAs is markedly higher than in Scotland, suggesting some underlying risks not captured in this analysis. Risk-based surveillance is an effective and efficient method that reduces the cost by testing fewer animals whilst improving/maintaining the level of disease detection. A full evaluation of the best strategy would entail a cost-benefit analysis considering the impacts of all changes in testing rates, impact on herd restrictions, and risk of onward transmission, and more subtly, changes in surveillance that may have unintended consequences for farmer behaviour. While such analyses lie outside the scope of this paper, the results presented here, specifying the purely epidemiological benefits of risk-based surveillance, are the essential groundwork for such a broader task.

## Supplementary material

For supplementary material accompanying this paper visit https://doi.org/10.1017/S0950268817001935. click here to view supplementary material
