# Supplementary material for: Risk-based strategies for surveillance of tuberculosis infection in cattle for low-risk areas in England and Scotland
Source: Epidemiol Infect. 2017 Dec 6;146(1):107–18. doi: 10.1017/S0950268817001935 (PMC5851039; doi:10.1017/S0950268817001935)
Supplement: Supplementary file 1 [file S0950268817001935sup001.docx]

**Epidemiology and Infection**

**Risk-based strategies for surveillance of tuberculosis infection in cattle for low risk areas in England and Scotland**

L.C.M. Salvador, M. Deason, J. Enright, P.R. Bessell, R.R. Kao

**Supplementary Material**

**Supplementary text**

**S1. Cattle movements from high-risk areas (HRAs) as a predictor of bovine Tuberculosis (bTB) breakdowns**

*S1.1. Source data*

The data used in this study were derived from the Cattle Tracing System (CTS) movement data (RADAR), and the SAM’s records of bTB tests and incidents (Defra). The CTS database contains records of all inwards and outward cattle movements in Great Britain. As the risk of transmission of bTB at markets is expected to be low, we used a version of the dataset with markets stripped from movements: a movement from *farm_1_* to a market to *farm_2_* is recorded as a movement from *farm_1_* to *farm_2_*. With these data a variety of risk ratings were generated for each cattle movement from 2007 to 2010 and a risk rating for each farm based on the ratings of their inward movements for each year of the study period.

*S1.2. Risk ratings*

*S1.2.1. Movement risk ratings*

Let be a movement if farm to farm on data . Let be the number of animals moved on that day between those farms. For each movement, four different risk ratings were calculated:

- if farm is in 1-year testing county, 0 otherwise.
- where is the number of animals moved if has had a bTB breakdown within  ten years previous to , 0 otherwise.
- where is the number of animals moved and is the date of the most recent breakdown at if has had a bTB breakdown within ten years previous to , 0 otherwise.
- where is the date of the most recent breakdown at if has had a bTB breakdown within ten years previous to , 0 otherwise.

*S1.1.2. Farm risk ratings*

For farm in year :

- ,where is within two years previous to .
- , where is within two years previous to .
- , where is within two years previous to .
- , where is within two years previous to .

*S1.3. Stump classifiers*

A stump classifier is a simple threshold classifier. Given a threshold and a set of rated examples (in this case, farms with risk ratings), all examples were classified with ratings above the threshold as positive, and below the threshold as negative. For a given rating measure, a threshold was chosen, and everything below the threshold was classified as non-risky and everything above as risky. At each threshold, the results were compared with bTB breakdowns and the number of true positives and false positives were recorded. The curve joining these points is a Receiver Operator Characteristic (ROC) curve and each curve represents the analysis of one risk rating for one given year. ROC curves are commonly used in machine learning and radiology [1] to assess the performance of a classifier or diagnostic test. A perfect classifier would be in the upper left hand corner of the plot, whereas a non-informative random classifier would be on the diagonal line from bottom left to top right corner. In order to understand if movements from high incidence areas are good predictors of bTB breakdowns, different risk ratings for all eligible herds were calculated as stump classifiers in three geographical areas: low risk areas (LRAs), Scotland and all United Kingdom. A set of distinct stump classifiers thresholds was used and compared with the actual bTB breakdowns where the number of true and false positives in each one of the three areas was recorded.

In Figure S5, the curves of the low incidence areas in England are more concentrated on the upper left side of the graph, therefore the prediction of breakdowns in these areas using movements as risk factors is better than in Scotland or in the rest of the UK. The prediction of breakdowns in the rest of the UK is also better than in Scotland, since the ROC curves are more concentrated in the upper left corner. The variation between risk ratings values for Scotland is large, which indicates that some risk ratings predict breakdowns, while others (that are very close to the diagonal line) do not predict more than a random guess. In summary, stump classifiers evaluated the role of different movement types as risk factors for spread, and broadly speaking showed that movements from high incidence areas to low incidence areas in England were a good (better than random) predictor of risk (Figure S5).

**S2. Underlying risk factors for bTB incidents**

*S2.1. Data extraction from SAM and CTS databases*

The ‘Herd table’ from SAM includes data on herd type (which was separated in the categories according to Table S1). The ‘Test table’ from SAM includes data on all herd test types and their results, while the ‘Incidents table’ has data on herds that recorded new confirmed breakdowns. Holdings that are registered but do not have animals, that are not active for the entire study period, or that hold herds of different types are not considered in this analysis.

Data on births, deaths, imports and movements of individual cattle to each holding were extracted from the British Cattle Movement System (BCMS) Cattle Tracing System Database (CTS). Only herds that have animals (through birth or movement) were considered in the analysis resulting in 13327 retained herds from 15570 low risk animal holdings or land keepers registered during that period. Since Scotland has been considered a low risk area since well before the study period, and considered officially bTB-free since 2012, all eligible and active herds in Scotland that follow the characteristics above have been retained (a total of 10145 herds from 11145 animal holdings or land keepers registered).

*S2.2. Multivariate logistic mixed models*

Multivariate logistic mixed models were formulated to assess candidate risk factors in which the outcome was 1 if holding *i* in county *j* recorded at least one confirmed incident of bTB in year k, and 0 otherwise. Data from the year 2007 was used to determine the number of movements and Irish imports for the following year, but models were constructed using data for LRAs only between 2008 and 2013. Both year and county were evaluated as random effects in order to estimate the mean distribution of the outcome in every permutation of year and county, as well as to control for any influence that these values may have on estimating holding level prevalence of infection. The following fixed effects were considered:

1. The mean herd size for each year. The yearly mean was computed by averaging the monthly size per holding (provided by APHA), as calculated from the number of births, deaths and recorded cattle movements off/onto individual holdings. Herd sizes were categorized to reflect population distributions in England (1-50, 51-100, 101-200, 201-350, 351-500 and >500 animals for LRAs) corresponding to the categories used in the RADAR 2008 Cattle Book [2]), and in Scotland (1-100, 101-350, 351-500 and >500).
2. The consolidated herd types found in the SAM database. These types were reduced to beef, dairy, finishing (beef animals that are ready for slaughter), suckler (a herd of cattle composed of dams and their young calves up to the point of weaning) and store (beef animals brought for finishing, normally well-grown animals of up to two years of age, which have been reared on one or more farms, and then are sold, either to dealers or other farmers) as in Table S1. An additional group including herds that did not fall into the aforementioned categories (“Other:) was excluded due to the poor representation of these holdings in the bTB incidence database.
3. Irish imports expressed as a binary response indicating whether a holding imported any Irish cattle in the previous year.
4. High-risk movements expressed as the number of batches (a batch being identified as animals moving between the same pair of holdings in the same direction in the same day) of cattle (categorised as 0, 1-10, and >10) arriving from HRAs to LRAs in the previous year for LRAs or as a binary response indicating whether a holding had received batches of cattle from HRAs in the previous year for Scotland.

*S2.3. Model evaluation*

Models were evaluated using the AIC model score. To account for possible temporal and spatial clustering, year and parish were fit as random effects. The best fit models were chosen by back-fitting the listed fixed effects, forward-fitting the random effects and finally back-fitting the fixed effects once more to recheck the selected variables. This rechecking is done because the inclusion of random effects could render certain fixed effects insignificant [3]. The model was reduced and just the predictors that were significant at p<0.05 were included in the final model. Interactions and associations between variables were also explored. The significant fixed effects are presented as odds ratios (Odds) with 95% confidence intervals. The fitted values extracted from the most significant model correspond to the probability of the herd becoming infected in each year of the study ($p_{intro}$).

**S3. Risk-based surveillance model**

*S3.1. Elements of risk and detection*

The risk-based surveillance scenarios were modelled considering the following elements of infection risk and detection:

a) The size of herds, with larger herds are at a greater risk of infection;

b) The proportion of the herd’s total stock that is sent to slaughterhouse, with holdings that send fewer stock to the slaughterhouse requiring more surveillance. This proportion is calculated based on the total number of cattle slaughtered in the previous year divided by the herd size. Only animals that are moved directly from the farm to slaughterhouse, or from farm to market and then direct to slaughterhouse are considered;

c) Whether the herd receives animals from HRAs in GB or Ireland. It is assumed that herds that receive animals from these areas have higher chances of getting infected.

*S3.2. Model assumptions*

The risk-based surveillance scenarios were modelled considering the following assumptions:

1) All herds that were consistently low risk during the entire study period and that only had one herd type could be included in the analysis;

2) All animals of a herd are tested, even if some animals are not tested under RHT; 3) All the other testing schemes (such as slaughterhouse, pre- and post-movement, tracings, contiguous herd, etc.) would continue; and that the sensitivity and specificity of the SICCT does not vary from herd to herd.

*S3.3. Point score system for risk-based scenarios*

During the risk-based simulations, the level of risk of each herd determines the testing interval of each herd, which is based on a point system (0 points=no testing, 1 point=4 year testing, 2 points=2 year testing, 3 points=annual testing). All risk-based scenarios allow for exemption from testing for herds with very low risk, except scenarios 7-8, which are more conservative and require the testing of all herds. In these scenarios, herd size was accounted as the most important risk factor and all the herds below a certain threshold value (scenario 7: herd size ≤ 350, scenario 8: herd size ≤ 500) are tested every four years, while the ones above the threshold are tested every year.

**Supplementary Figures**

Figure S1. **Change of national bTB testing regimes between 2009 and 2013 according to Defra/AHVLA.** High-risk areas have expanded over time reducing the areas of low risk, and a two-early testing buffer (edge area) was created between 2009 and 2011. In 2013, the edge areas were expanded and the testing regime changed to only two time windows (1-year testing for high risk and edge areas, and 4-year testing for areas of low risk.


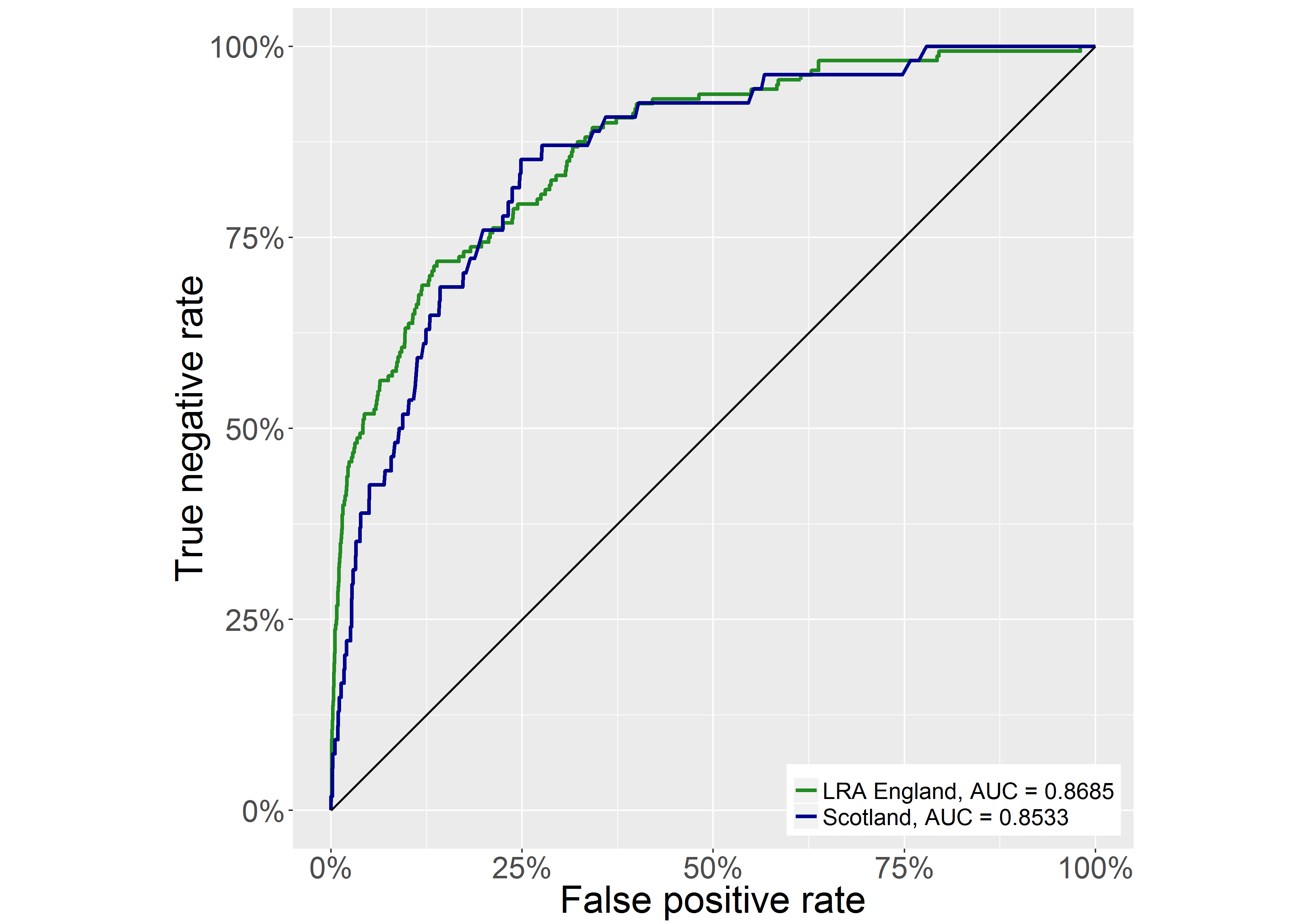


Figure S2. **Receiver Operating Characteristics curve for the model fit for the risk factors in LRAs and in Scotland.** The area under the curve (AUC) is presented next to each respective model in the legend. The solid black line represents a model that assigns the binary response at random.

**A**


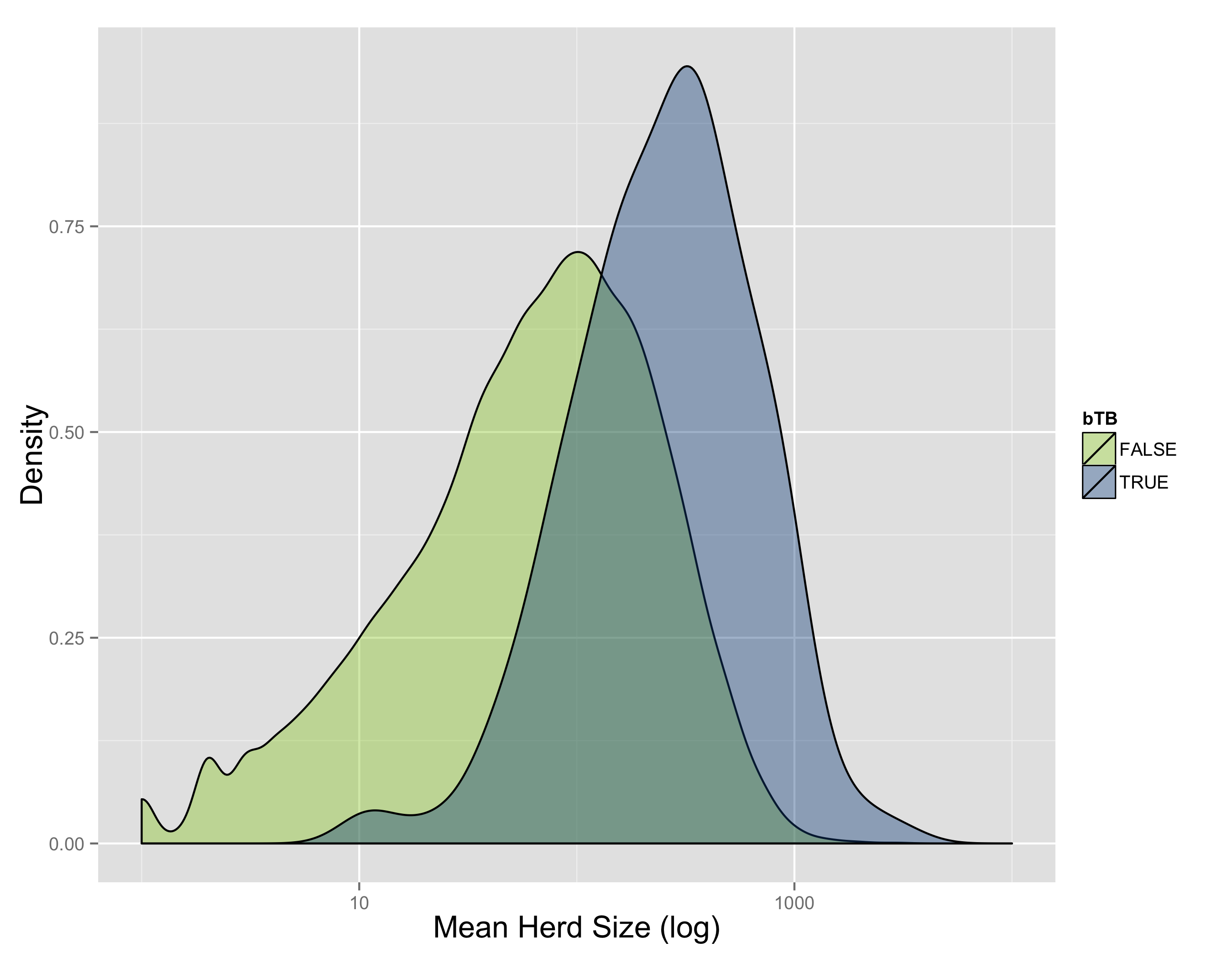


**B**


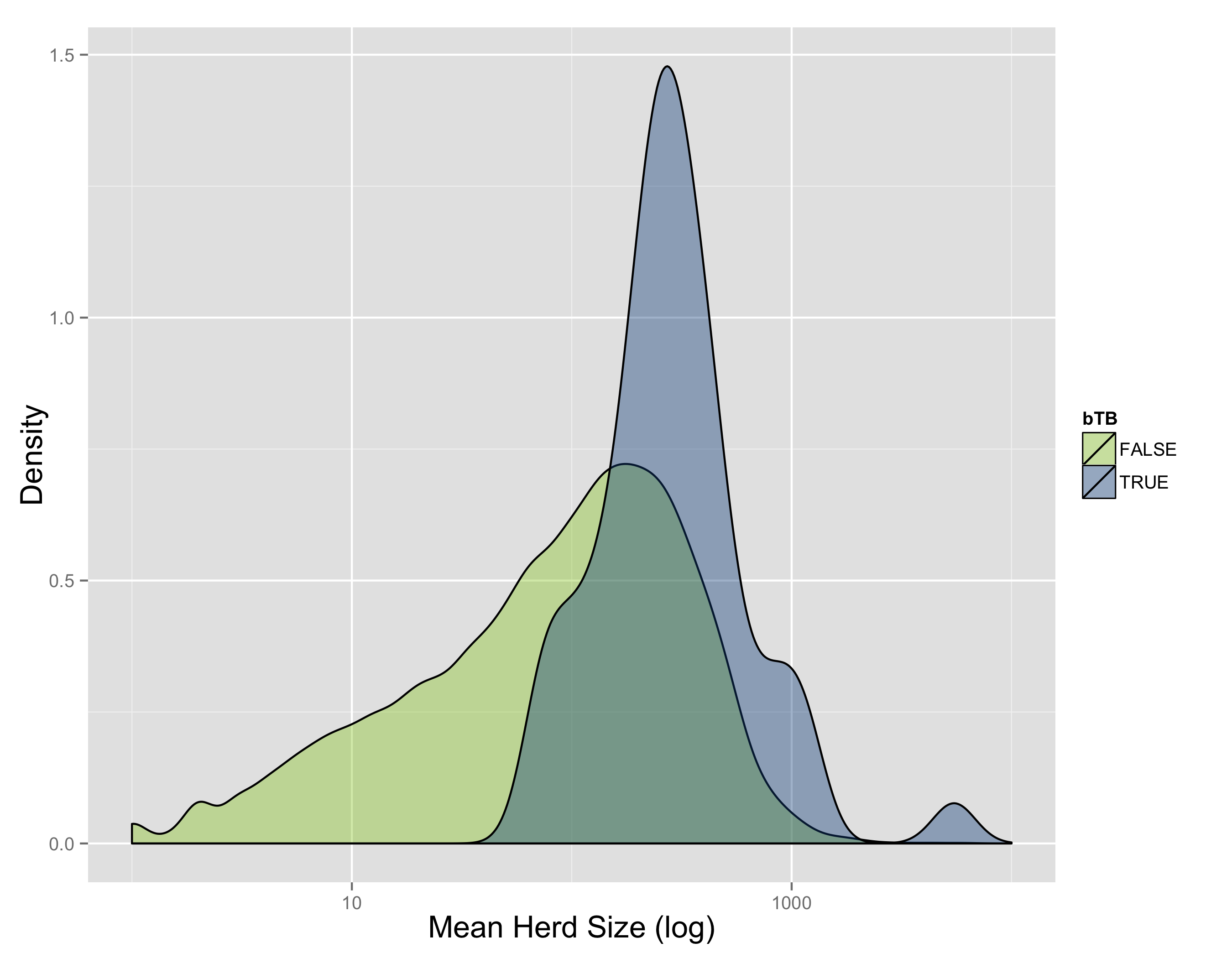


Figure S3. **Mean herd size versus number of herd breakdowns**. Density distributions of the mean herd size of farms with at least one breakdown (in blue) and without breakdowns (in green) during 2008-2013 in (A) LRAs and in (B) Scotland. The x-axis is in log scale.

**A**


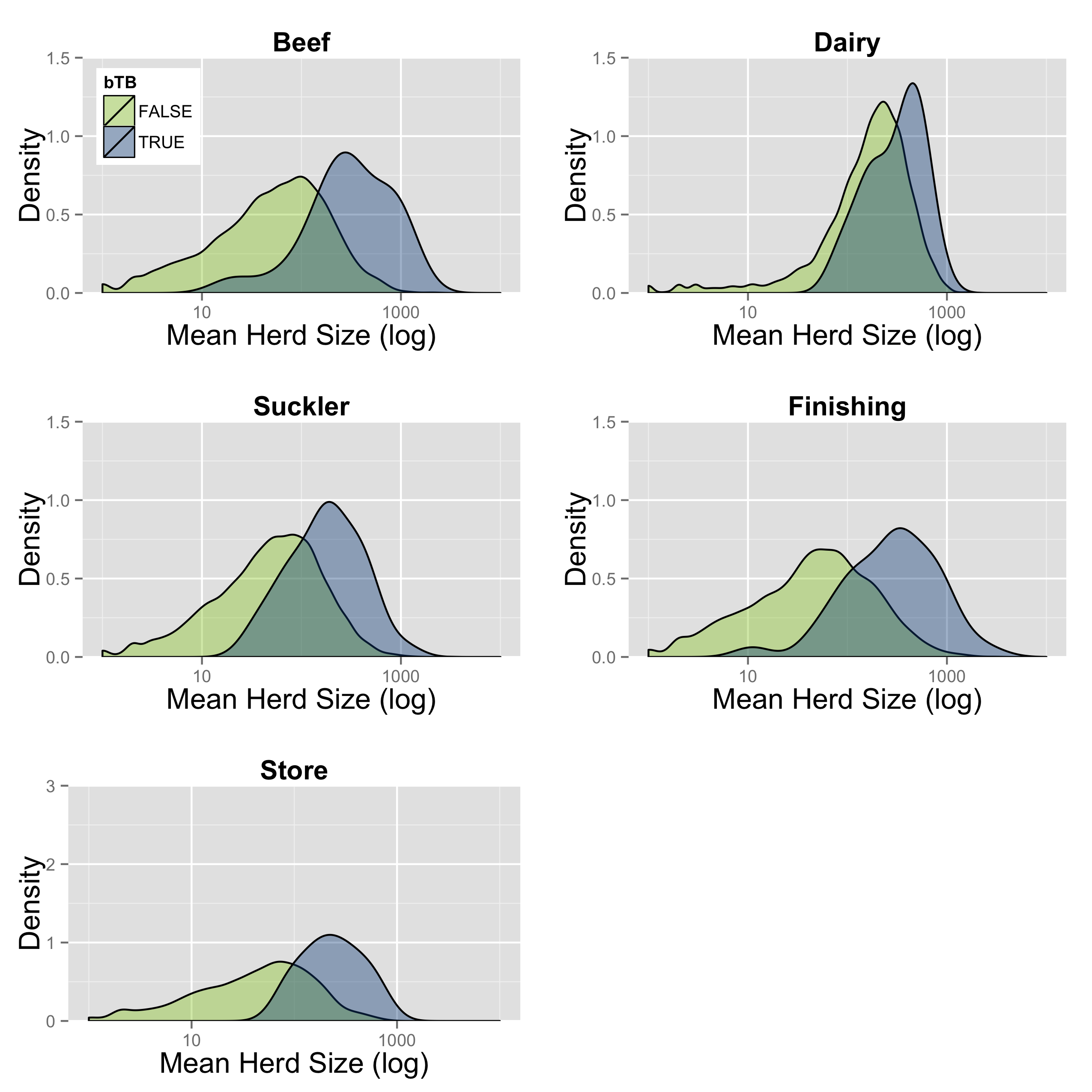


**B**


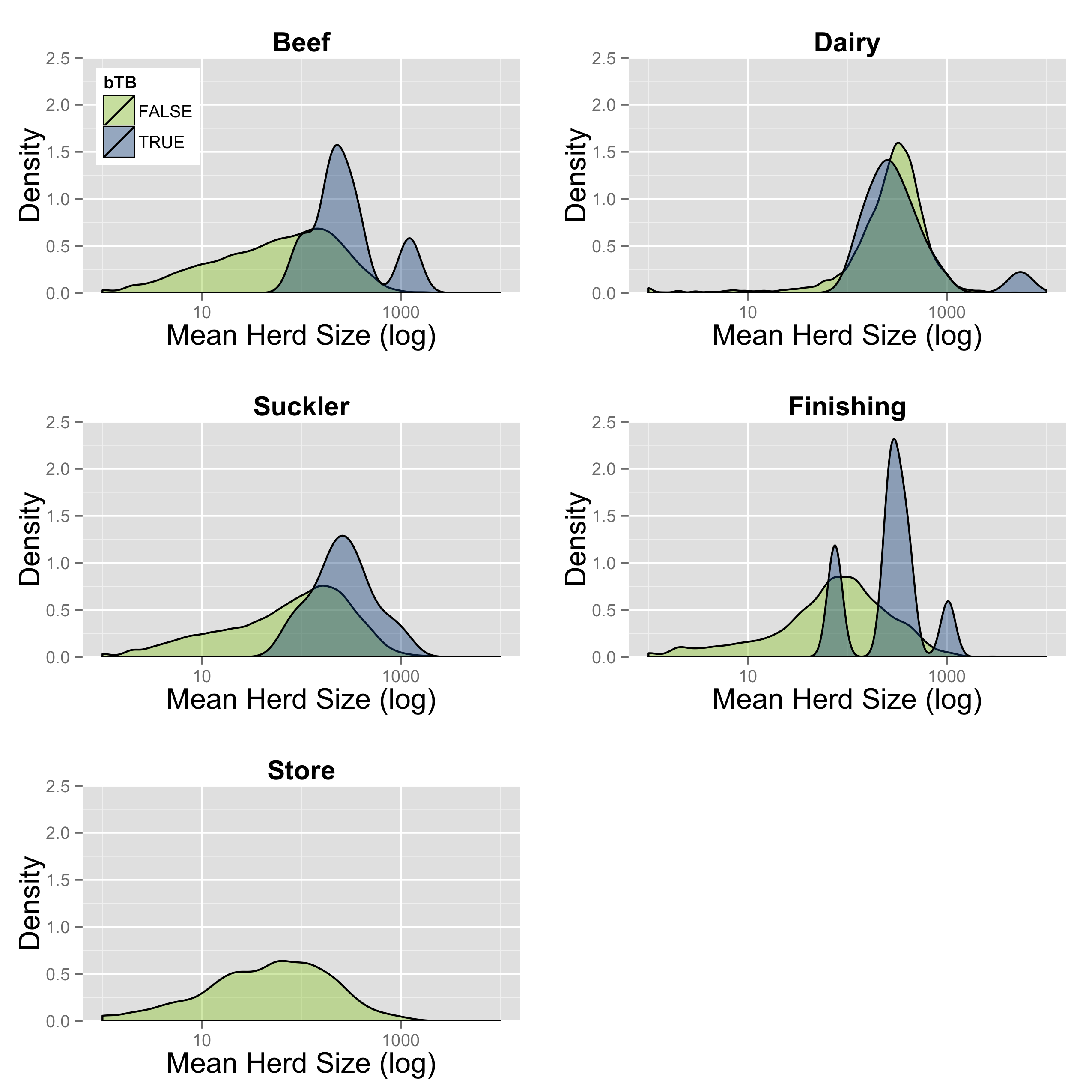


Figure S4: **Mean herd size versus number of herd breakdowns per herd type.** Density distributions of the mean herd size of farms with at least one breakdown (in blue) and without breakdowns (in green) separated by herd type in (A) LRAs and in (B) Scotland during 2008-2013.

**
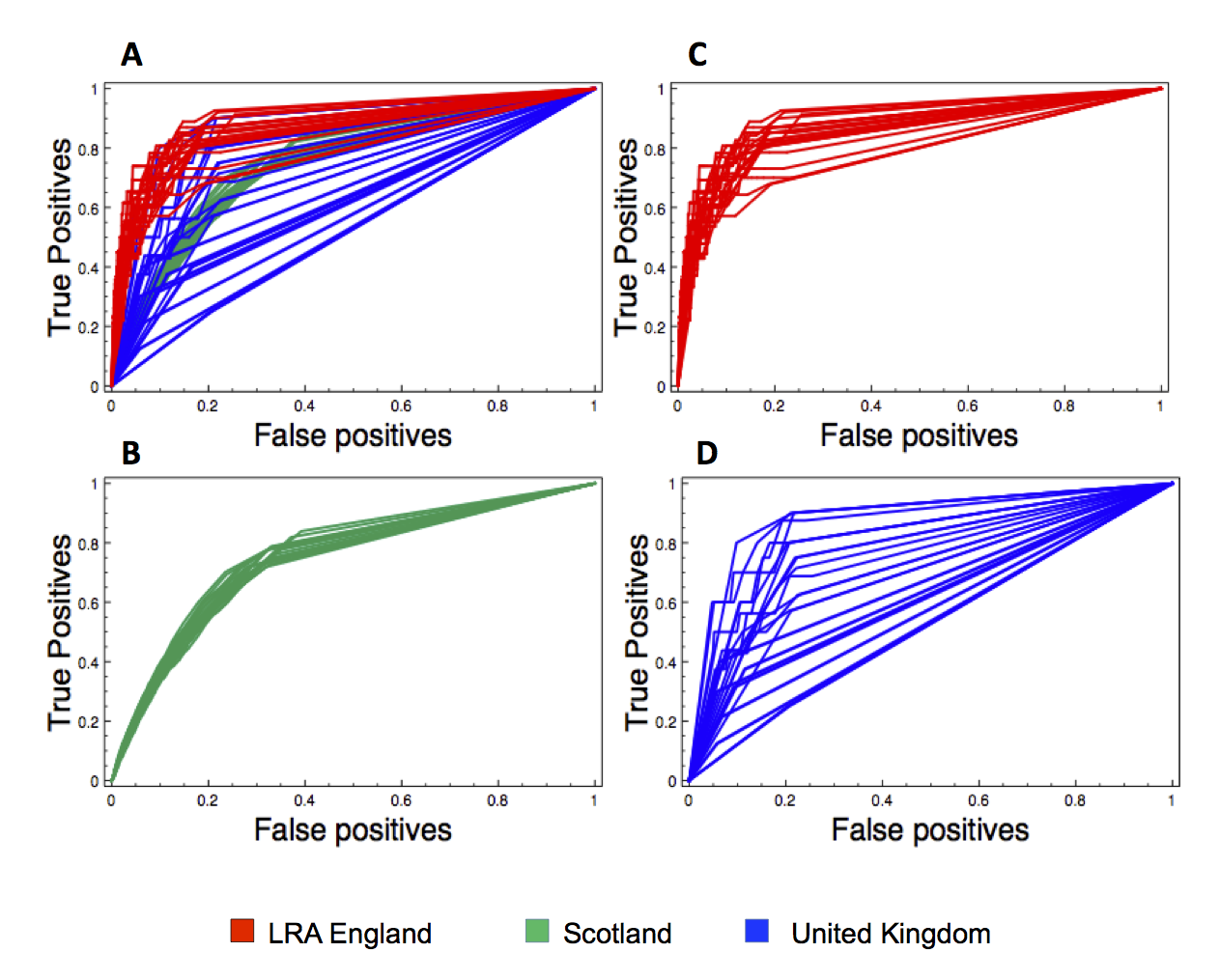
**

Figure S5: **Cattle movements as risk factors in LRA England, Scotland and UK.** Receiver Operator Characteristic (ROC) curves from all simulation runs of the stump classifier analysis during the time period 2007-2010 for all the three areas together (A), for United Kingdom (B), for LRA England (C), and for Scotland (D).

**A**

**B**

Figure S6. **Herd type versus** **the probability of freedom from infection.** Boxplot showing the herd probability of freedom from infection with the slaughterhouse only model by herd type in LRAs (A) and in Scotland (B) during 2009-2013. Probability of freedom of infection is defined as 1-probability of undetected infection. Box widths represent the proportion of data in each herd type category. The y-axis has been truncated for clarity.

**A**

**B**

Figure S7. **Movements to slaughterhouse versus herd size.** Scatterplots showing the squared root of mean herd size per herd against the squared root of the mean number of animals moved to slaughter in LRAs (A) and in Scotland (B) in 2009-2013 broken down by herd type as listed in SAM’s database. The axes have been truncated for clarity. There is a positive relationship between herd size and number of movements to slaughter with distinct clustering of finishing and dairy herds. Dairy herds are both larger and typically have fewer per capita movements to slaughter compared to finishing herds.

**A**


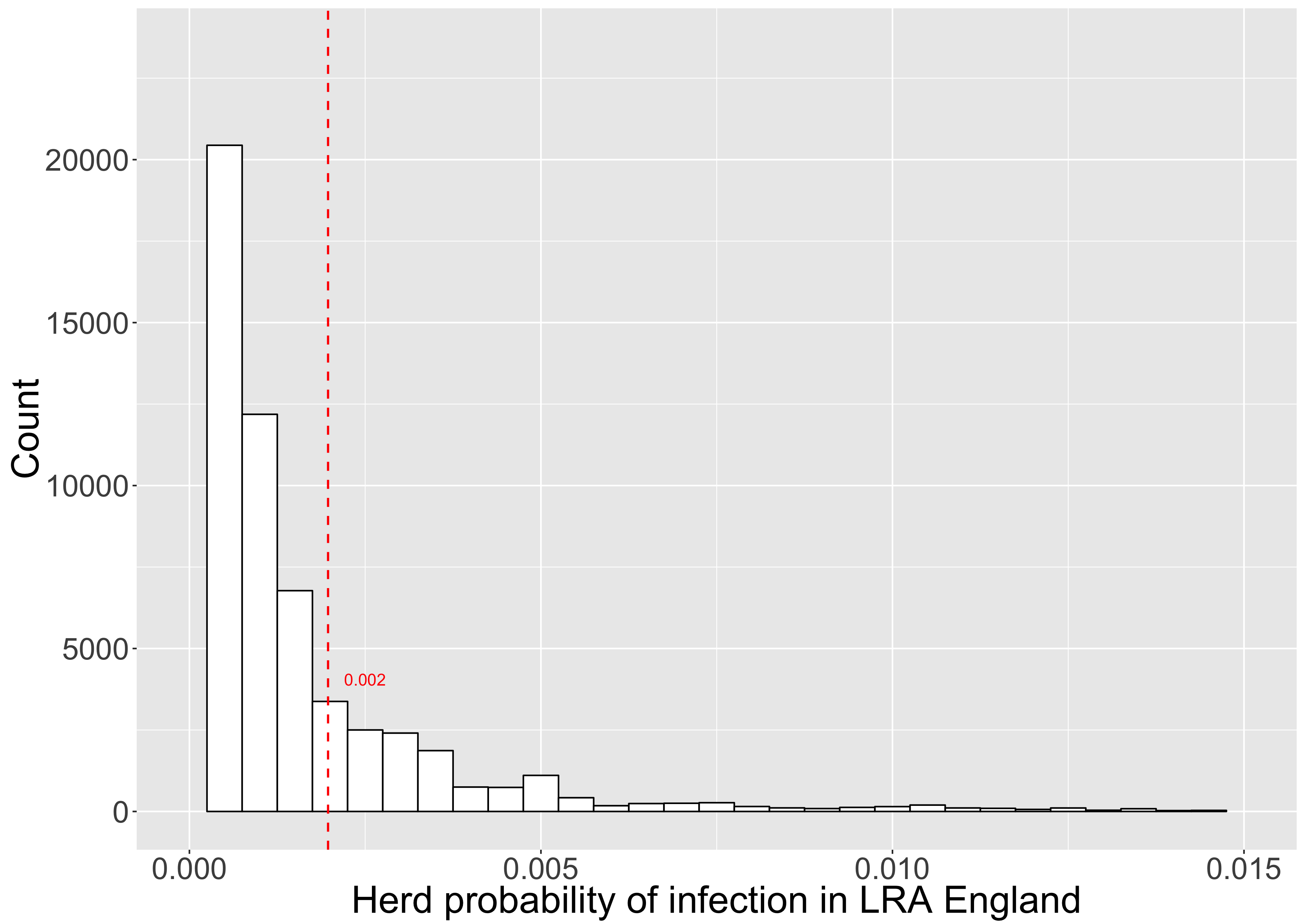


**B**


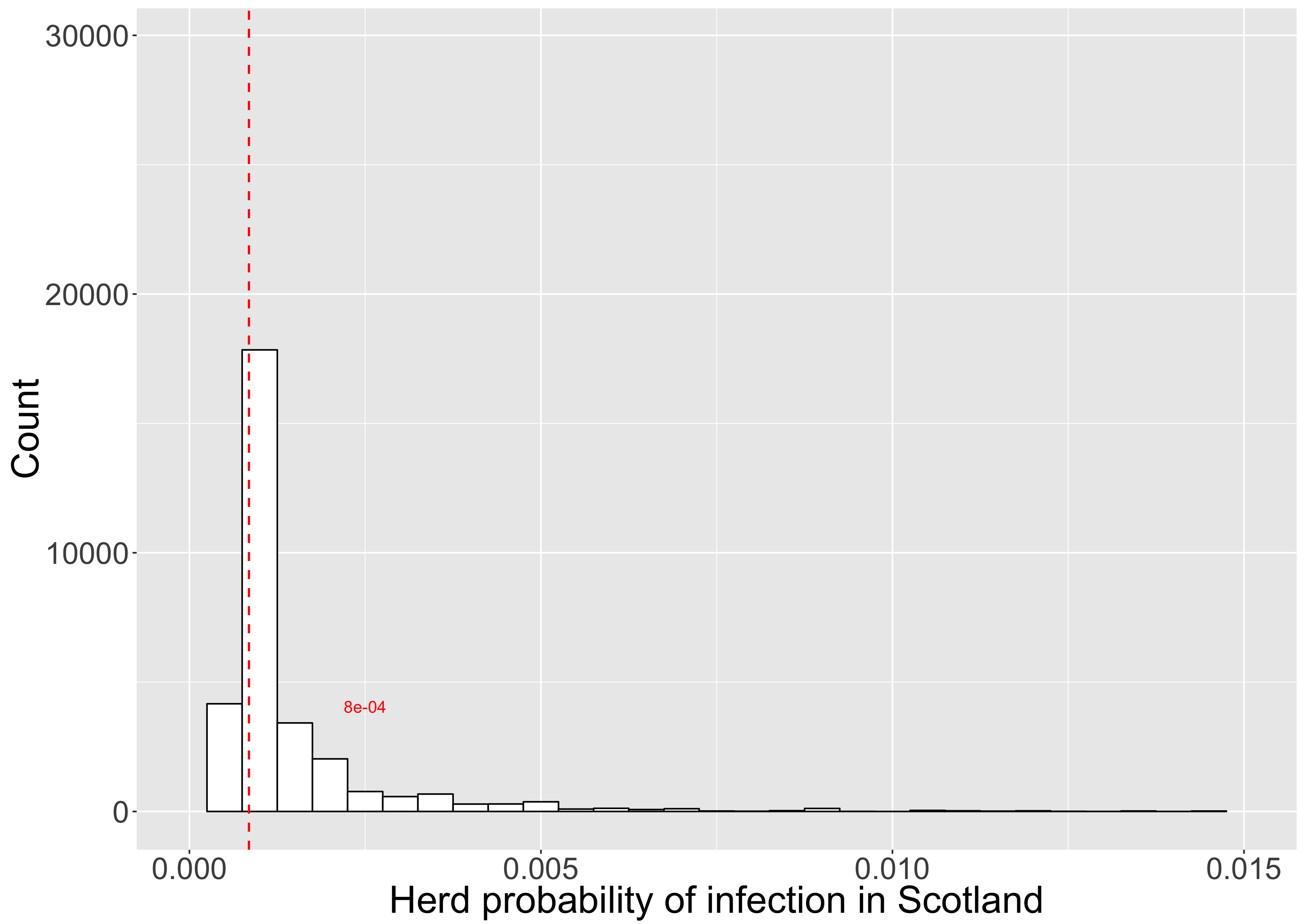


Figure S8. **Histogram of the herd probability of infection estimated by the GLMs used for LRA England (A) and for Scotland (B).** The dashed red vertical lines represent the mean values of the herd probability of infection. The axes were truncated for clarity.

**A**

**
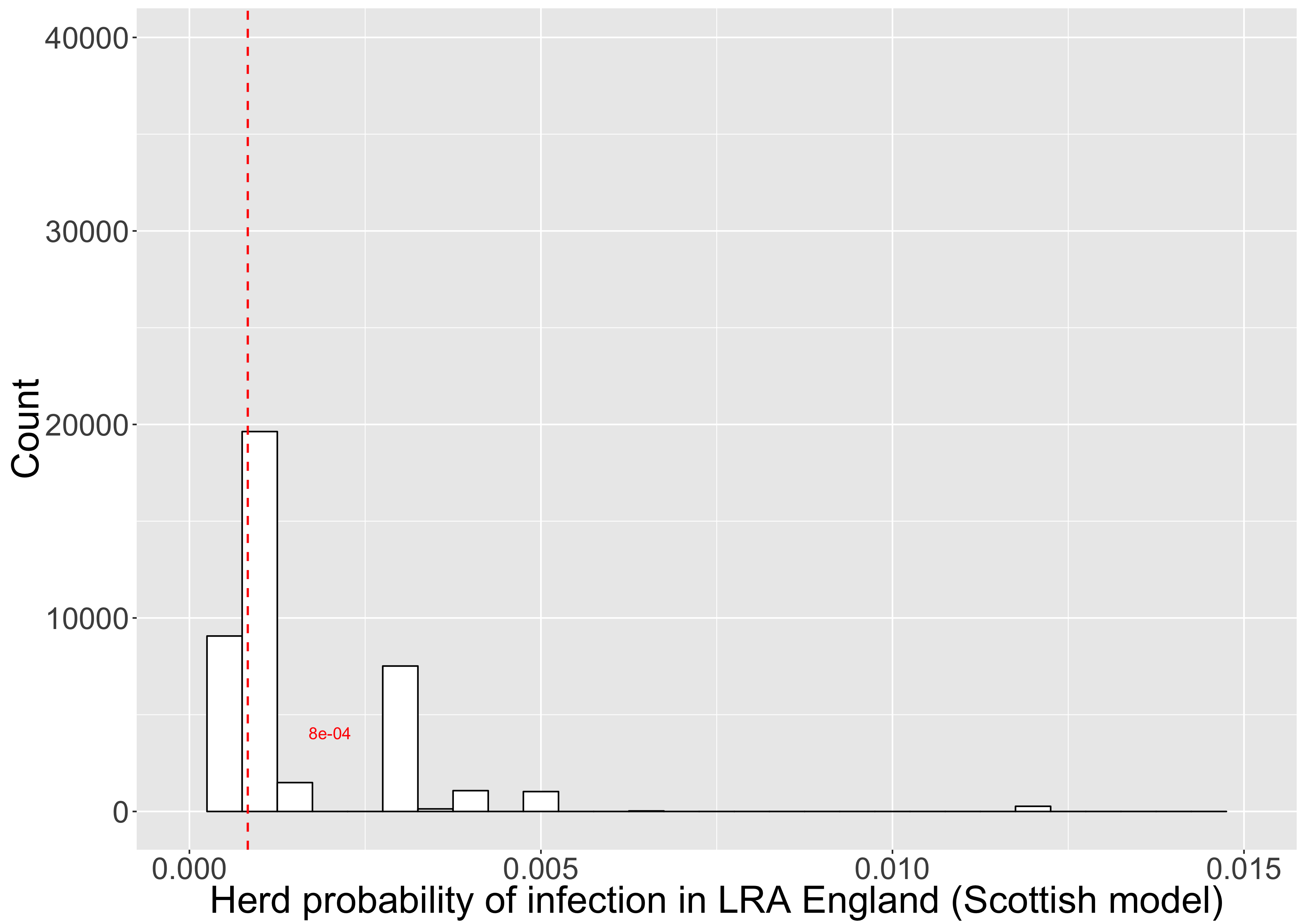
**

**B**

**
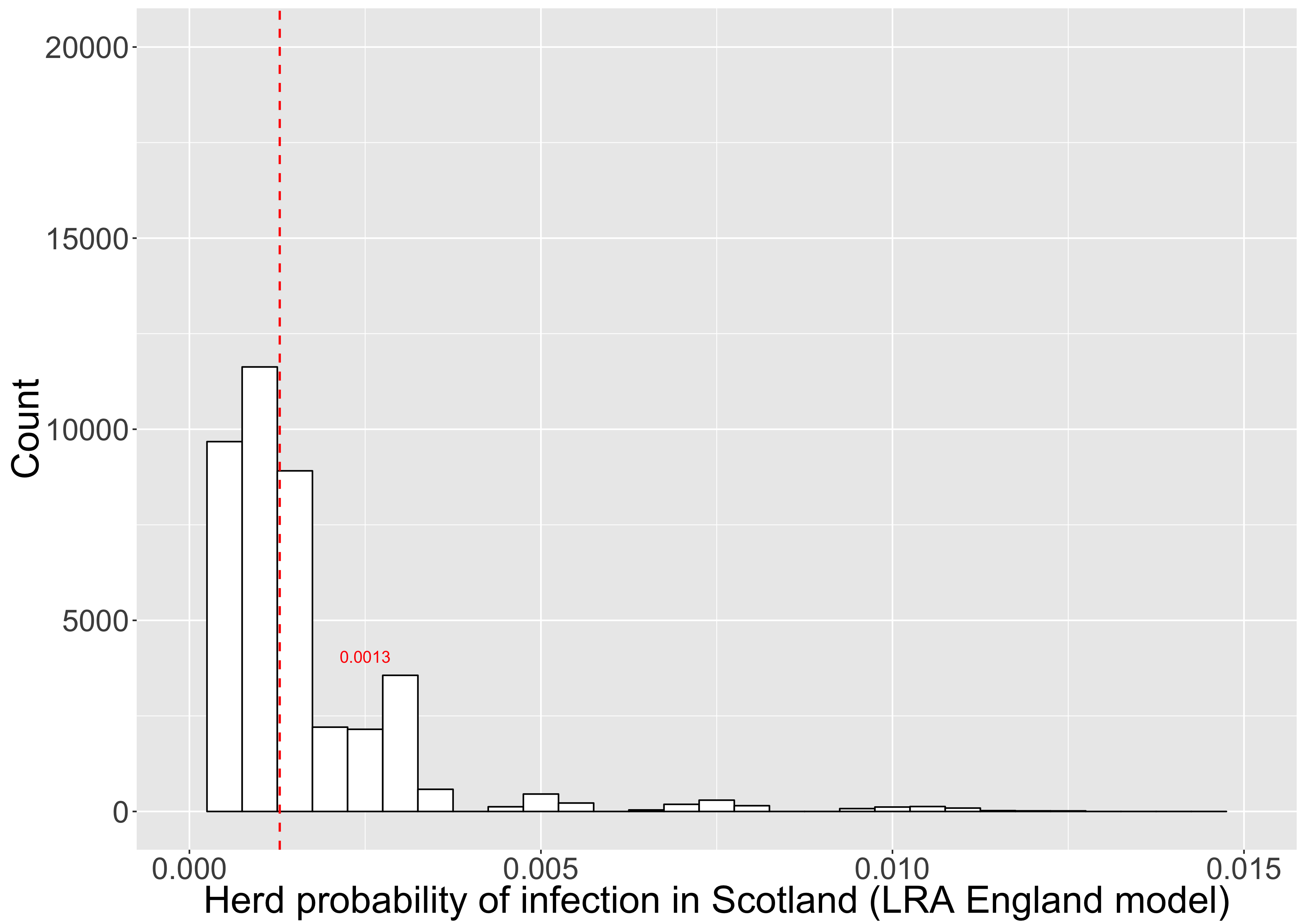
**

Figure S9. **Histogram of the herd probability of infection estimated by the GLMs used for herds in LRA using the Scottish model predictor (A) and for herds in Scotland using the LRA model predictor (B).** The dashed red lines represent the means values of the herd risk of infection. The axes were truncated for clarity.

**Supplementary Tables**

| **Herd type** | **Specific herd categories** |
| --- | --- |
| Beef | Beef, BEEF, Beef Bull Hirer, Beef Dealer, Beef, Heifer Rearer, BISON, Bull Beef, Dealer, DEALER, Meat |
| Dairy | Dairy, DAIRY, Dairy Bull Hirer, Dairy Dealer, Dairy Heifer Rearer, DAIRY P/R, DAIRY/DEAL, Domestic, Producer, Producer/Caterer, Producer/Processor, Producer/Retailer, Producer/Wholesale |
| Store | Stores |
| Finishing | Finishing |
| Suckler | Suckler, BEEF SUCKLER |
| Other | All the other categories in SAM’s database |

Table S1. **Herd type categories.** Characterization of herd types in their sub-categories according to SAM’s database. The ‘Other’ category is not used in our analyses.

**A**

| **Predictor** | **Unit** | **Odds (95% CI)** | **Z value** | **Pr(>|Z|)** |
| --- | --- | --- | --- | --- |
| Intercept | 0 | 0 | 23.83 | <0.001 |
| Herd size | 1-50 | 1 | - | - |
|  | 51-100 | 3.74 (1.7-8.2) | 3.31 | 0.001 |
|  | 101-200 | 5.43 (2.60-11.34) | 4.50 | <0.001 |
|  | 201-350 | 8.25 (3.94-17.28) | 5.59 | <0.001 |
|  | 351-500 | 12.30 (5.55, 27.23) | 6.19 | <0.001 |
|  | >500 | 17.02 (7.99, 36.27) | 7.34 | <0.001 |
| Batches from HRAs | 1 | 0 | - | - |
|  | 1-10 | 3.38 (2.26, 5.07) | 5.92 | <0.001 |
|  | >10 | 18.27 (11.75, 28.41) | 6.19 | <0.001 |
| Irish imports | False | 1 | - | - |
|  | True | 2.34 (1.36, 4.02) | 3.06 | 0.002 |

**B**

| **Predictor** | **Unit** | **Odds (95% CI)** | **Z value** | **Pr(>|Z|)** |
| --- | --- | --- | --- | --- |
| Intercept | 0 | 0 | -17.44 | <0.001 |
| Herd size | 1-100 | 1 | - | - |
|  | 101-350 | 5.23 (2.88-23.58) | 3.93 | <0.001 |
|  | 351-500 | 11.31 (3.43, 37.33) | 3.93 | <0.001 |
|  | >500 | 14.75 (4.55, 47.75) | 4.49 | <0.001 |
| Batches from HRAs | False | 1 | - | - |
|  | True | 3.20 (1.62, 6.30) | 3.35 | 0.001 |
| Irish imports | False | 1 | - | - |
|  | True | 4.22 (2.17, 8.22) | 4.23 | <0.001 |

Table S2. **Risk factor model results for LRA in England and Scotland.** Results of a mixed logistic regression model that compute the herd risk of infection to determine the risk factors associated with bovine Tuberculosis breakdowns in LRAs (A) and in Scotland (B) during 2008-2013. Odds ratio, 95% confidence intervals express the contribution of each one of the significant risk factors, Z-score and respective p-values are used to determine whether a certain predictor variable is significant or not.

|  |  | **Herds from Scotland** | | **Herds from LRA England** | |
| --- | --- | --- | --- | --- | --- |
| **Category** | **Unit** | **<=0.025** | **>0.025** | **<=0.025** | **>0.025** |
| Herd Size | 0-50 | 0.335 | 0 | 0.411 | 0 |
|  | 51-100 | 0.160 | 0.013 | 0.201 | 0.033 |
|  | 101-200 | 0.207 | 0 | 0.202 | 0.085 |
|  | 201-350 | 0.161 | 0.324 | 0.115 | 0.326 |
|  | 351-500 | 0.071 | 0.216 | 0.038 | 0.198 |
|  | >500 | 0.065 | 0.446 | 0.033 | 0.388 |
| Herd Type | Beef | 0.270 | 0.135 | 0.215 | 0.143 |
|  | Dairy | 0.121 | 0.081 | 0.215 | 0.033 |
|  | Finishing | 0.087 | 0.189 | 0.163 | 0.613 |
|  | Store | 0.033 | 0.027 | 0.072 | 0.071 |
|  | Suckler | 0.489 | 0.568 | 0.335 | 0.139 |
| Irish Imports | False | 0.968 | 0.378 | 0.991 | 0.832 |
|  | True | 0.032 | 0.622 | 0.009 | 0.168 |
| Batches from HRAs | 0 | 0.959 | 0 | 0.770 | 0 |
|  | 1-10 | 0.041 | 0.203 | 0.217 | 0.009 |
|  | >10 | 0 | 0.797 | 0.013 | 0.991 |

**Table S3.** Table showing the proportion of herds in Scotland and in LRA England that fall in each category for low (<=0.025) and high (>0.025) herd probabilities of infection. The herd probabilities of infection were computed using the LRA England model predictor and the 0.025 value was chosen as the threshold that separates a well mixed number of LRA England and Scottish herds from mainly LRA England herds (Figure 4).

**References**

[1] A. J. Hanley and J. B. McNeil, “The Meaning and Use of the Area under a Receiver Operating Characteristic (ROC) Curve,” *Radiology*, vol. 143, pp. 29–36, 1982.

[2] DEFRA-Publications, “The RADAR Cattle Book 2007,” 2007.

[3] J. J. Dziak, D. L. Coffman, S. T. Lanza, and R. Li, “Sensitivity and specificity of information criteria,” *PeerJ Prepr.*, 2015.
